# Contrast-free T1 mapping at 3T can characterize chronic myocardial infarctions with high diagnostic accuracy

**DOI:** 10.1186/1532-429X-16-S1-P205

**Published:** 2014-01-16

**Authors:** Avinash Kali, Ivan Cokic, Richard Tang, Hsin-Jung Yang, Behzad Sharif, Eduardo Marbán, Debiao Li, Daniel S Berman, Rohan Dharmakumar

**Affiliations:** 1 Biomedical Imaging Research Institute, Cedars-Sinai Medical Center, Los Angeles, California, USA; 2 Department of Biomedical Engineering, Northwestern University, Evanston, Illinois, USA; 3 Department of Biomedical Engineering, University of California, Los Angeles, California, USA; 4 Cedars-Sinai Heart Institute, Cedars-Sinai Medical Center, Los Angeles, California, USA; 5 Department of Medicine, University of California, Los Angeles, California, USA; 6 Department of Radiology, Northwestern University, Chicago, Illinois, USA

## Background

Characterizing myocardial infarctions (MIs) on the basis of LGE CMR requires gadolinium infusion, which poses limitations in certain patient populations and imaging workflow. We hypothesized that T1 differences between MI and remote territories at 3T would enable reliable characterization of chronic MI.

## Methods

Canines (n = 29) underwent CMR at 7 days (acute) and 4 months (chronic) following reperfused MIs at 3T (n = 19) and 1.5T (n = 10). Contrast-free T1 maps (MOLLI; 8 TIs with 2 inversion blocks of 3+5 images; minimum TI = 110 ms; ΔTI = 80 ms; TR/TE = 2.2/1.1 ms) and LGE images (IR-prepared FLASH; TI optimized to null remote myocardium; TR/TE = 3.5/1.75 ms) were acquired. MI location, size and transmurality were determined using Mean+5SD criterion relative to remote myocardium. T2 maps (T2-prepared SSFP; T2 preparation times = 0, 24 and 55 ms; TR/TE = 2.8/1.4 ms) were acquired to compare acute and chronic MIs. Commonly used imaging parameters were slice thickness = 6 mm and spatial resolution = 1.3 × 1.3 mm 2. Histological validation was sought to confirm the presence of replacement fibrosis within the chronic infarct zones.

## Results

**Figure 1:**
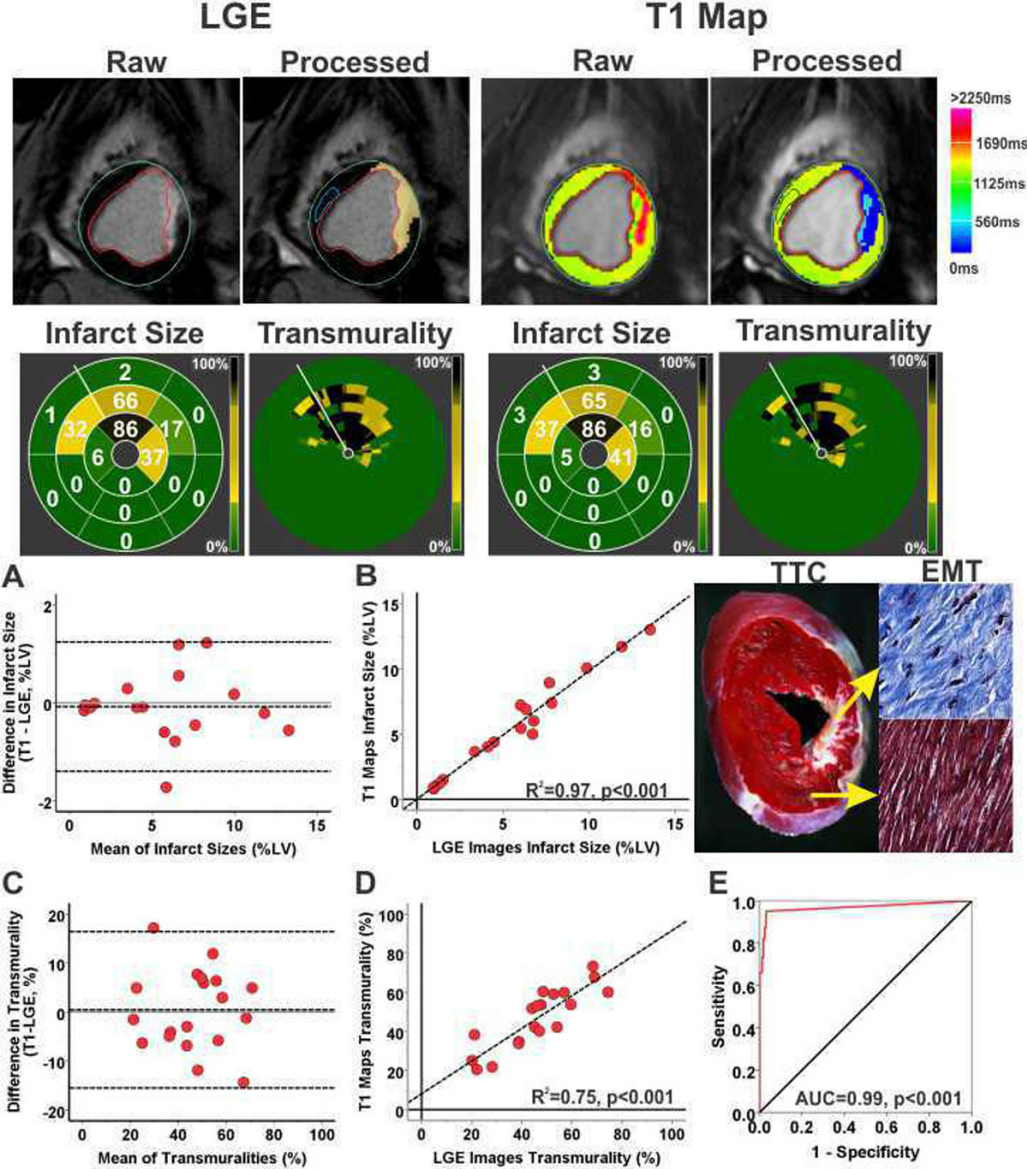
**Representative slice-matched LGE images and contrast-free T1 maps acquired from a canine imaged at 3T (4 months post MI) are shown**. Post-processed images delineating the MI territories using Mean+5SD criterion relative to remote myocardium are also shown. Hypointense core of iron deposition within hyperintense MI zone on T1 maps was manually included in the analysis (highlighted light blue pixels on the processed images). AHA 17-segment bulls-eye plots showed excellent correlations between LGE images and T1 maps for measuring infarct volume (IV) and transmurality (IT). Ex-vivo TTC and EMT staining showed extensive replacement fibrosis within MI (top panel), but not in remote myocardium (bottom panel). Strong agreement and correlation were observed between LGE images and T1 maps for measuring chronic IV (Bias = -0.08 ± 0.68% (panel A) and R2 = 0.97 (panel B)) and IT (Bias = 0.45 ± 8.14% (panel C) and R2 = 0.75 (panel D)) at 3T. Area under the curve for detecting chronic MI at 3T using T1 maps was 0.99 (panel E).

Contrast-free T1 maps and LGE images of a representative mid-ventricular slice, along with AHA 17-segment bulls-eye plots depicting the MI size and transmurality acquired from a canine scanned imaged 4 months post-MI at 3T are shown in Figure 1. Bland-Altman plots, linear regression plots and receiver-operating characteristic curve comparing T1 maps and LGE images for measuring infarct volume (IV, %LV) and transmurality (IT) in the chronic phase at 3T are also shown. At 3T, T1 maps and LGE images were not different for measuring IV (5.6 ± 3.7% vs. 5.5 ± 3.7%; p = 0.61) and IT (44 ± 15% vs. 46 ± 15%; p = 0.81) in the chronic phase, but were significantly different in the acute phase (IS: 13.3 ± 8.4% vs. 11.6 ± 6.8%, p = 0.007 and IT: 64 ± 19% vs. 56 ± 17%, p = 0.007). At 1.5T, IV and IT were significantly underestimated by T1 maps relative to LGE images during acute (IS: 9.4 ± 5.6% vs. 15.5 ± 9.4%, p < 0.001 and IT: 59 ± 5% vs. 76 ± 6%, p < 0.001) and chronic phases (IS: 2.1 ± 1.2% vs. 4.8 ± 1.8%, p < 0.001 and IT: 47 ± 7% vs. 66 ± 9%, p < 0.001). At 3T and 1.5T, T1 values of the MI remained elevated in both acute (3T: p < 0.001; 1.5T: p < 0.001) and chronic phases (3T: p < 0.001; 1.5T: p = 0.037) compared to remote myocardium (Table 1). At both 3T and 1.5T, relative to the remote myocardium, T2 values of the MI were elevated in the acute phase (p < 0.001 for both cases), but were not different in the chronic phase (3T: p = 0.19, 1.5T: p = 0.55). Ex-vivo TTC and Elastin-modified Masson's Trichrome (EMT) stainings (Figure 1) confirmed extensive replacement fibrosis within the MI territories at 4 months post MI. Sensitivity and specificity of contrast-free T1 maps at 3T for detecting chronic MIs were 95% and 97%, respectively.

**Table 1:** T1, T2 and LGE signal intensity characteristics of acute and chronic myocardial infarction at 1.5T and 3T

Field Strength	3T				1.5T			
**Time Post-MI**	**Day 7**		**Month 4**		**Day 7**		**Month 4**	

**Tissue Type**	**Remote**	**Infarcted**	**Remote**	**Infarcted**	**Remote**	**Infarcted**	**Remote**	**Infarcted**

T1 (ms)	1230 ± 63	1563 ± 154	1257 ± 138	1485 ± 139	924 ± 72	1104 ± 108	976 ± 80	1060 ± 116

T2 (ms)	46 ± 4	64 ± 9	44 ± 4	46 ± 3	50 ± 4	69 ± 5	49 ± 5	51 ± 6

%Change in T1 with respect to Remote	26 ± 8		19 ± 7		14 ± 8		12 ± 6	

%Change in LGE signal intensity with respect to Remote	728 ± 484		790 ± 513		376 ± 192		409 ± 163	

Sensitivity of Contrast-Free T1 maps	94%		95%		84%		58%	

Specificity of Contrast-Free T1 maps	94%		97%		74%		78%	

## Conclusions

Contrast-free T1 maps at 3T can determine the location, size and transmurality of chronic MIs with high diagnostic accuracy.

## Funding

This work was supported in parts by grants from National Heart, Lung and Blood Institute (HL091989) and American Heart Association (SDG 0735099N).

